# Focused ultrasound therapy in movement disorders: management roadmap toward optimal pathway organization

**DOI:** 10.3389/fneur.2024.1356310

**Published:** 2024-02-26

**Authors:** Sara Rinaldo, Roberto Cilia, Valentina Leta, Mariarosaria Gammone, Nico Golfrè Andreasi, Fabiana Colucci, Arianna Braccia, Roberta Telese, Marco Fusar Poli, Vincenzo Levi, Luigi Michele Antonio Romito, Francesco Ghilemetti, Elena De Martin, Maria Luisa Fumagalli, Francesca Epifani, Sara Prioni, Paolo Amami, Sylvie Piacentini, Antonio Emanuele Elia, Grazia Devigili, Vittoria Nazzi, Elisa Francesca Maria Ciceri, Mario Stanziano, Marina Grisoli, Valentina Caldiera, Marisa Catotti, Francesco DiMeco, Giacomina Clara Moreschi, Roberto Eleopra

**Affiliations:** ^1^Health Professions Management, Fondazione IRCCS Istituto Neurologico Carlo Besta, Milan, Italy; ^2^Parkinson and Movement Disorders Unit, Department of Clinical Neurosciences, Fondazione IRCCS Istituto Neurologico Carlo Besta, Milan, Italy; ^3^Parkinson's Centre of Excellence at King's College Hospital and King's College London, London, United Kingdom; ^4^Neuropsychology Unit, Fondazione IRCCS Istituto Neurologico Carlo Besta, Milan, Italy; ^5^Functional Neurosurgery Unit, Department of Neurosurgery, Fondazione IRCCS Istituto Neurologico Carlo Besta, Milan, Italy; ^6^Health Department, Fondazione IRCCS Istituto Neurologico Carlo Besta, Milan, Italy; ^7^Neuroradiology Unit, Department of Diagnostic and Technology, Fondazione IRCCS Istituto Neurologico Carlo Besta, Milan, Italy; ^8^Imaging Radiology and Interventional Neuroradiology, Department of Neurosurgery, Fondazione IRCCS Istituto Neurologico Carlo Besta, Milan, Italy; ^9^Department of Neurological Surgery Fondazione IRCCS Istituto Neurologico Carlo Besta, Milan, Italy; ^10^Department of Oncology and Hemato-oncology, University of Milan, Milan, Italy; ^11^Department of Neurological Surgery, Johns Hopkins Medical School, Baltimore, MD, United States

**Keywords:** clinical pathways, MRgFUS, focused ultrasound, thalamotomy, essential tremor, Parkinson’s disease

## Abstract

MRI-guided focused ultrasound (MRgFUS) lesioning is an innovative, safe and effective treatment which provides an innovative development in the field of minimally invasive stereotactic neurosurgery. Based on the application of focused ultrasound energy under full MR planning and thermal imaging control, unilateral lesioning of the thalamus, subthalamic nucleus, and globus pallidus is indicated for the treatment of movement disorders, including essential tremor, Parkinson’s disease, and dystonia. We started to apply this technique in February 2019 for the treatment of patients with movement disorders. The authors developed a diagnostic therapeutic care pathway, which is herewith proposed and applied as an explication of standard clinical practice in use. The project was the result of the application of different methods such as Health Technology Assessment (HTA), Strengths, Weaknesses, Opportunities and Threats analysis (SWOT) and Demin -Plan, Do, Check, Act (PDCA) cycle. The aim of this project was to standardize the MRgFUS diagnostic-therapeutic pathway (DTP), describe its application and the appropriateness of different phases (patient selection, intervention phase and follow-up). Here, we described in detail our experience in the DTP application from 2019 up to now in 610 patients with movement disorders.

## Introduction

Improving the quality of life of patients with movement disorders [including Parkinson’s disease (PD) and tremor syndromes such essential tremor (ET) and dystonia] is one of the most critical challenges due to their progressive motor and non-motor disability. Therefore, it is essential to implement an integrated and multidisciplinary approach that can reduce the impact of disability on patients’ quality of life; depending on the circumstances and stages of the disease, this may involve many professionals. The field of movement disorders management continues to evolve and change at a remarkable pace. Interventional therapies, including surgical options, are increasingly used globally to treat movement disorders, in addition to pharmacological and rehabilitative approaches (1–3).

Among interventional approaches, magnetic resonance-guided focused ultrasound (MRgFUS) ablation therapy is a non-invasive modality requiring neither craniotomy nor skin incision for the treatment of ET, unilateral tremor in PD or dystonia and neuropathic pain (4). It immediately appeared necessary and indispensable to structure a pathway for patients with movement disorders eligible for interventional therapies to offer them the best personalized option based on international guidelines and expert consensus and on the availability of healthcare institute in terms of expertise, facilities, technology, staff available. The comprehensive definition of diagnostic-therapeutic pathways (DTPs) provided during the 2005 Consensus Meeting in Slovenia describes them as a methodology aimed at sharing decision-making processes and organization of care for a specific group of patients during a well-defined period of time. According to the European Pathway Association (EPA), the purpose of DTPs is to increase the quality of care perceived and delivered, improving outcomes and promoting patient safety through the use of the right resources needed.

This manuscript aims to describe the standardized process and to show the results of the application of the DTP that we have used since 2019 targeted to patients with medication-refractory tremor, from the screening for eligibility to MRgFUS treatment to the long-term follow-up. We herewith report the development and application of a specific DTP starting from the identification of a model pathway, then continuing with the analysis of the actual working reality at the given historical moment, to the definition of an actual pathway, which is applicable in real-life and in the context of the specific institutional scenario, considering the environmental reality, skills, knowledge, experience, and competencies at the Foundation IRCCS Carlo Besta Neurological Institute, Milan, Italy (hereinafter referred to as ‘our Institute’) at the time when the path definition activities began.

The development of a DTP starts from a review of the current literature on assessments programs for interventional procedures in movement disorders, associated with a careful analysis of the existing operative and managerial reality at our Institute. The expected result was to establish a consistent basis for the development of a series of standardized and specific activities referring to the different phases of the DTP. The outcome was the development and application of a DTP embedded within an integrated process mapping for all the ‘interventional therapies’ available at our Institute.

## Materials and methods

Considering that both the technology and the MRgFUS procedure represented two novelties for our center, we deemed it appropriate to carry out a Health Technology Assessment (HTA) before starting to develop the pathway. It is a multidisciplinary process that evaluates the clinical, economic, organizational, social, ethical, and safety implications related to the introduction, diffusion, and use of health technologies.

The objective of the HTA analysis was to assess the actual and/or potential effects of technology, as well as the consequences that the introduction of the specific type of technology could have for the health care system, economy, and society. The evaluation of the effectiveness of health technology was conducted by employing a systematic review of literature, which is the most comprehensive and structured methodological tool.

### HTA: literature search

Essential and common elements of the methodological tool used were:

- the literature search, consistent with the research question;
- the selection of studies, based on the predefined inclusion and exclusion criteria;
- the critical analysis of the quality of the included studies and the synthesis of the data.

An analogous consideration was made in setting up the research for the analysis of the safety, organizational, ethical, and social aspects of the specific technology. The instruments were imprinted with the method used for the evaluation of effectiveness; additional and specific aspects were considered, such as the specialized resources to be consulted for information retrieval. As for the evaluation of the “economic” domain, the methodological approaches employed were:

- systematic review of economic studies;
- cost analysis/estimation;
- economic evaluation (with the formulation of an economic model);
- economic analysis (review/research of economic studies and from the economic evaluation).

Case series, observational studies, and randomized controlled trials on focused high-field ultrasound for the treatment of ET and tremor in PD were considered (5–8). Previous HTA research conducted in other countries was also evaluated (9).

### Analysis of strengths, weaknesses, opportunities, and threats

The SWOT Analysis was constructed through the classic matrix divided into four fields:

1. Strengths—Factors within the context to be enhanced;
2. Weaknesses—Limits to be considered;
3. Opportunities—Possibilities that are offered by the context and can provide opportunities for development;
4. Threats—Risks to be assessed and addressed because they could worsen and make a situation critical.

For this type of analysis, it is crucial to be specific circumscribing the object and being clear about the objective, because a generic analysis would be ineffective.

The ***advantages*** of such analysis can be summarized in three points:

1. The deep analysis of the context in which one acts made possible by the preliminary observation and collection of data and their skillful interpretation results in a timely delineation of strategies.
2. The continuous comparison between the needs of the organization and the strategies adopted leads to an enhancement of the effectiveness achieved.
3. It allows for a greater consensus on strategies if all parties involved in the intervention participate in the analysis.

The ***limitations*** associated with this type of analysis are the following ones:

1. risk of describing a too simplified reality.
2. its implementation requires a partnership context, which if not realized, runs the risk of a disconnect between the theoretical and the political-pragmatic plan.

### Diagnostic therapeutic pathway: working group definition and document drafting

In this DTP, a multidisciplinary and multi-professional team made up of personnel from different Operating Units (Parkinson and Movement Disorders Unit, Functional Neurosurgery Unit, Radiotherapy, Diagnostic and Interventional Neuroradiology, Intensive Care Unit, Neurophysiology Unit, Health Service, Neuropsychology Unit) were responsible for screening, treatment, and monitoring of patients undergoing MRgFUS at our Institute.

The work team consisted of all the professionals involved in the pathway: neurologist, neurosurgeon, radiotherapist, medical physics expert, anesthesiologist, neuroradiologist, clinical psychologist, radiology technician, neurophysiology technician, engineering support staff and administrative.

The DTP is intended as an explication of current practice in a specific institution, in a specific time and in a specific operative contest; it is not intended to be only a systematic review of the literature on the subject and a passive application of founded indications, but an adaptation of it to the existent work frame. In general terms, the DTP procedure verifies the appropriateness of patient selection, the intake of cases selected for the procedure, the stage of the intervention, and the short- and long-term follow-up of patients.

The pathway was developed as being applicable only to patients with ET and unilateral tremor in PD while being part of an operational structure with greater organizational complexity, which is that for advanced therapies in movement disorders.

**Figure 1:**
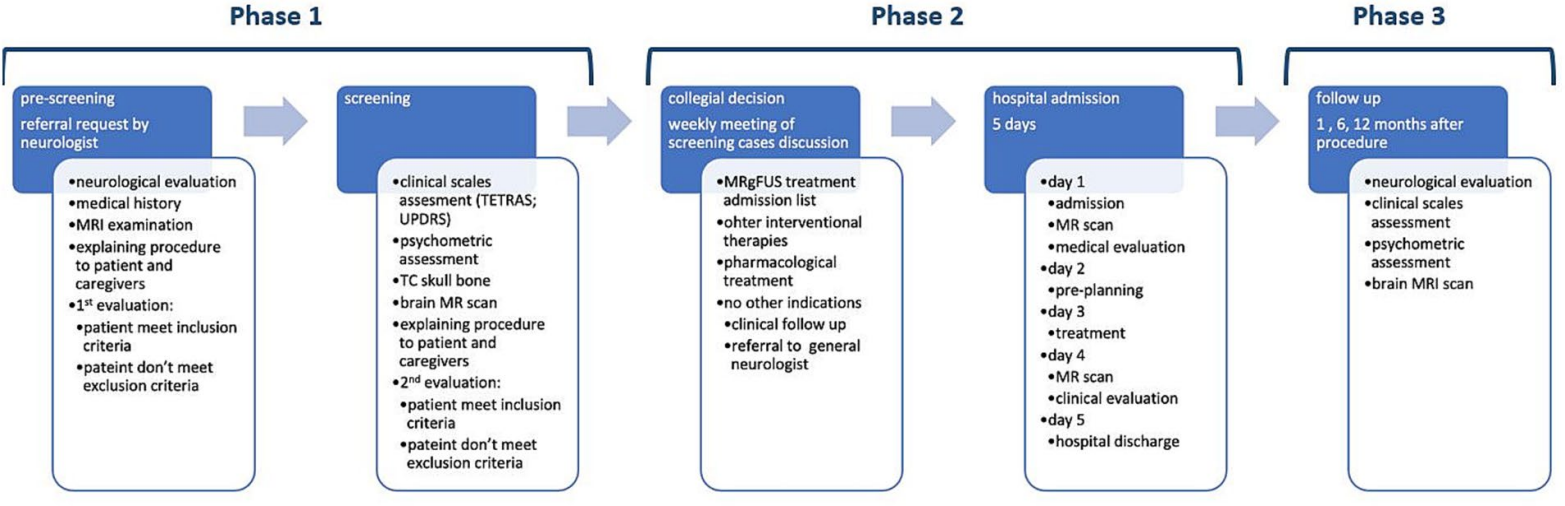
Diagnostic care pathway for the treatment of unilateral tremor with MR-guided high-field focused ultrasound.

Three essential phases characterize this DTP are summarized in Figure 1:

- Pre-Treatment Screening Phase (patient selection)
- Intra-hospital Phase (Surgical Procedure)
- Follow-up phase (post-treatment)

The DTP has been diffused through an educational process of all health professionals involved through training meetings and its publication on the Institute Intranet.

A table of responsibilities has been edited and made available in the document, thus that each operator identifies a person or operational unit to interface with. Several clinical studies have been designed and approved by the local Ethics Committee.

## Real-world application of the pathway: defining organizational strategy and management of communication

To optimize communication and the acquisition of useful information for work planning, we have activated and widespread a corporate e-mail address to which internal and external neurologists can contact to refer patients they consider to be candidates for MRgFUS. We developed patient-specific information pages, also made available on the Foundation’s website, with first-contact information about the procedure. It immediately appeared essential to adopt a waiting list system to ensure that patient’s access to the screening pathway is organized in a linear and orderly manner. Therefore, we implemented a database in which to enter, at time of referral, the personal data and specifications regarding pathology and indication for treatment and type of access (whether screening or first neurological pre-screening assessment).

The database works by color code: yellow means awaiting screening, red screening performed with negative results, and green screening performed with positive results, thus to be placed on the waiting list for admission. Once placed on the waiting list in our specific data base management system, the color changes to white.

The Pathway Coordinator manages the database, which is then shared with the neurologists who perform the screening assessments.

Different operative structures are involved in the development of the pathway, each providing necessary primary and secondary processes. Several professionals in many different areas and sectors, even physically separated, must be promptly informed about the work organization, thus it immediately appeared essential to activate an internal communication tool within the Foundation that would allow for a precise, rapid, and effective mass dissemination of work plans to share a weekly organization plan for outpatients.

A similar scheme is necessary for the pathway manager to set up the basic operations required for admission for surgery: verify the list of patients for admission for MRgFUS procedures, availability of beds for admission, availability of high-technology operating rooms, alert the neuroradiology and OR coordinators, verify the availability of disposables, alert administration secretariat for patient-call in time for admission with the possible discontinuation of drug therapies, when indicated.

The planned work-plan for outpatients performing clinical diagnoses and evaluations for screening and follow-up is necessary for pathway coordination to establish the sequence of examinations/visits to be completed in the correct order based on the patient’s clinical status, the time of examinations or visits (so that they are performed in the proper clinical timing and without overlapping of schedules).

Once the workflow has been defined, the Pathway Coordinator sends the plan to all the professionals involved. The work plan, sent the week before, reported identification of the outpatient clinic/diagnostic area where the patient will be assessed, type of examination/evaluation, clinical protocol to be applied (screening, follow-up, and timing), the reference Neuroradiology, blood tests and any rapid swab to be carried out in the screening area if the stay in the institute is for more than 4 h. The operating schedule is spread over 4 out of 5 working days.

To verify the process quality of the DTP procedures, the working team defined indicators for each of the three specific stages. The corresponding rationale accompanies each indicator:

- for *Phase 1 (pre-treatment/screening),* the indicator will be the ratio between the number of cases selected for MRgFUS and the number of cases proposed (total). Rationale: selecting the correct candidate reduces the risk of failure and/or complications. The target value per year is >0.6: the appropriateness of sending is considered adequate if at least 6 out of 10 subjects have an effective indication for treatment.
- for *Phase 2 (treatment),* to ensure intra-operative and post-operative complication monitoring, the indicator will be the Number of cases undergoing MRgFUS without complications/Total Number of cases treated. In this case, the Outcome will be the safety of the procedure. The target value per year is >0.85: the procedure is considered adequately safe if at least 8 out of 10 subjects have no major side effects and/or adverse events.
- for *Phase 3 (follow-up),* to ensure monitoring of efficacy and long-term complications, the indicator will be the number of cases followed up 1 year after MRgFUS / total number of cases treated. The target value per year is >0.6: the level of clinical and instrumental assessment after treatment is considered adequate if at least 6 out of 10 treated subjects perform follow up visits in the 12 months following the procedure.

## Results

### HTA analysis

Evaluation results in the *clinical* domain:

- MRgFUS neurosurgery is an effective and generally safe treatment option for moderate to severe, drug refractory ET.
- It provides a treatment option for people unsuitable for invasive neurosurgery and offers a non-invasive option for all people considering neurosurgery.
- Patients not eligible or not accepting invasive neurosurgery (e.g., deep brain stimulation), MRgFUS lesioning is cost-effective compared to best medical therapy.
- In individuals eligible for invasive neurosurgery, MRgFUS may be one of several reasonable options.
- Patients with ET who underwent MRgFUS neurosurgery reported positive experiences. They appreciated the fact that it was a non-invasive procedure and reported a substantial reduction in tremor that resulted in an improvement in their quality of life.

Evaluation results in the *non-clinical* domain:

- The funding of MRgFUS neurosurgery for the treatment of moderate to severe, drug refractory ET at the Institute has been partly public and partly private. The economic investment is certainly significant, but the burden of disease estimates for PD and ET are higher.
- The treatment of tremor has a low care burden and an equally low cost in terms of consumables, with a recognized DRG equal to a craniotomy.

### SWOT analysis

Once the issue has been assessed from the point of view of health technology, one must think about the objective while simultaneously considering both internal and external variables: the SWOT analysis. Being specific is critical to this type of analysis: circumscribe the object and be clear about your objective, because a generic analysis would be ineffective.

Table 1 describes the SWOT analysis related to MRgFUS in movement disorders, to identify in depth all contingent factors and carry out an effective cross-reading of them.

**Table 1:** SWOT analysis related to MRgFUS in movement disorders.

**STRENGTHS** ***(Factors within the context to be enhanced)*** Solid group (long-standing collaboration) Opportunities to grow New knowledge Conviction Valid arguments Need to reflect on the DTP Comparison Training Experience Skills Flexibility and ability to confront Willingness for change	**WEAKNESSES** *(Limits to be considered)* Little time to carry out activities and in addition the project Dispersion of energy Clinical/organizational duplication Unready organization (conservatism) Inconsistency in actions and different messages to patients Lack of communication “Sacrifice” and tiredness Lack of concrete motivation Use of computers Long-established habits
**OPPORTUNITIES** ***(Possibilities that are offered by the context and can provide opportunities for development)*** Growth for the group, more dialog Social benefit (fewer hospital admissions) Optimization in budget management Improved forecasting requirements Greater well-being for patients More precise organization Greater actual and perceived safety Computer use Directing management of screening and planning procedures Optimisation of hospital bed management	**THREATS** ***(Risks to be assessed and addressed, because they could worsen and make a situation critical)*** Failure of the project Confusion Tiredness Conflicts Opposition to changes Incompetence Non-adherence to the project Physician-centered and not patient-centered view Disorganization Lack of confidence

Evaluated the context in all his relevant aspects considering the prospect to effectively start the screening pathway, the working group defined shared clinical criteria in inclusion/exclusion from the procedure. Table 2 reported the main grounds considered in deciding whether to proceed with treatment.

**Table 2:** Inclusion and exclusion criteria for MRgFUS treatment of unilateral tremor in PD and ET.

**Inclusion criteria** Diagnosis of ‘Essential Tremor’ resistant to at least 2 medications targeting tremor, with medium to severe disability - TETRAS scale (10) Diagnosis of clinically established ‘Parkinson’s disease’ predominantly unilateral tremor’ (11), who meet the following criteria: MDS-UPDRS-III scale (12) score ≥ 20 in OFF therapy Maintain stable medical therapy during the 30-day pre-procedure period Age > 18 years and ability to provide informed consent Ability to communicate their symptoms or distress during the procedure
**Exclusion Criteria** About patient with diagnosis of clinically established ‘Parkinson’s disease’: Hoehn and Yahr scale modified to ON therapy greater than 3. Atypical Parkinsonism (multisystem atrophy, progressive supranuclear palsy, corticobasal syndrome); Secondary Parkinsonism (drug-induced, vascular, normal-pressure hydrocephalus, etc). Previous CNS surgery including Deep Brain Stimulation; General exclusion criteria: Clinical Dementia according to the according to MDS criteria (13) or DSM-V (14); Unstable psychiatric disorders, defined as active and uncontrolled, such as: depression, psychosis, delirium, hallucinations or suicidal ideation, severe mood disorders such as to have required hospitalization in psychiatric settings, electroconvulsive therapy, or Transcranial Magnetic Stimulation in the previous 12 months; Contraindications deducible from ‘neuropsychological evaluation’: Subjects with a history of alcoholism or drug addiction Presence of significant cognitive impairment (MoCA ≤21) Serious cardiological pathologies such as: Unstable angina pectoris in therapy Recent IMA (within the previous 6 months) Severe congestive cardiomyopathy (FE < 40) Unstable cardiac arrhythmias Atrial arrhythmias not well controlled Severe arterial hypertension (not well controlled with medical therapy) Anticoagulant therapy (TAO or NAO) or anti-aggregants. Note: MRgFUS lesioning can be carried out in patients who can tolerate an adequate withdrawal of therapy (at least 7 days before the procedure) in accordance with the most recent guidelines on anticoagulant therapy (15). Known risk factors for intra- and post-operative bleeding, such as: documented and certain coagulopathy; platelet count <100,000/mmc. Severe chronic renal insufficiency (glomerular filtrate <30 mL/min) or on dialysis. Positive history of hemorrhagic or ischemic stroke in the previous 6 months or with MRI images suggestive of ‘cerebral amyloidosis’ Drug-resistant epilepsy Brain tumor or evidence of significant damage in the MRgFUS target areas. Intra-cranial aneurysms or intracranial arteriovenous malformations (AVMs). Contraindications to standard MRI, including those with implanted metallic devices, cardiac pacemakers/defibrillators, neurostimulators, shunts/stents, or other metallic implants in the brain. Severe claustrophobia, which cannot be managed with medication. Weight (kg) above the upper limit of what is allowed on the MRI table or who cannot be placed on the scanner. Patients who are unable to tolerate prolonged supine position during the procedure

### Real-world application of the pathway: results

**Figure 2:**
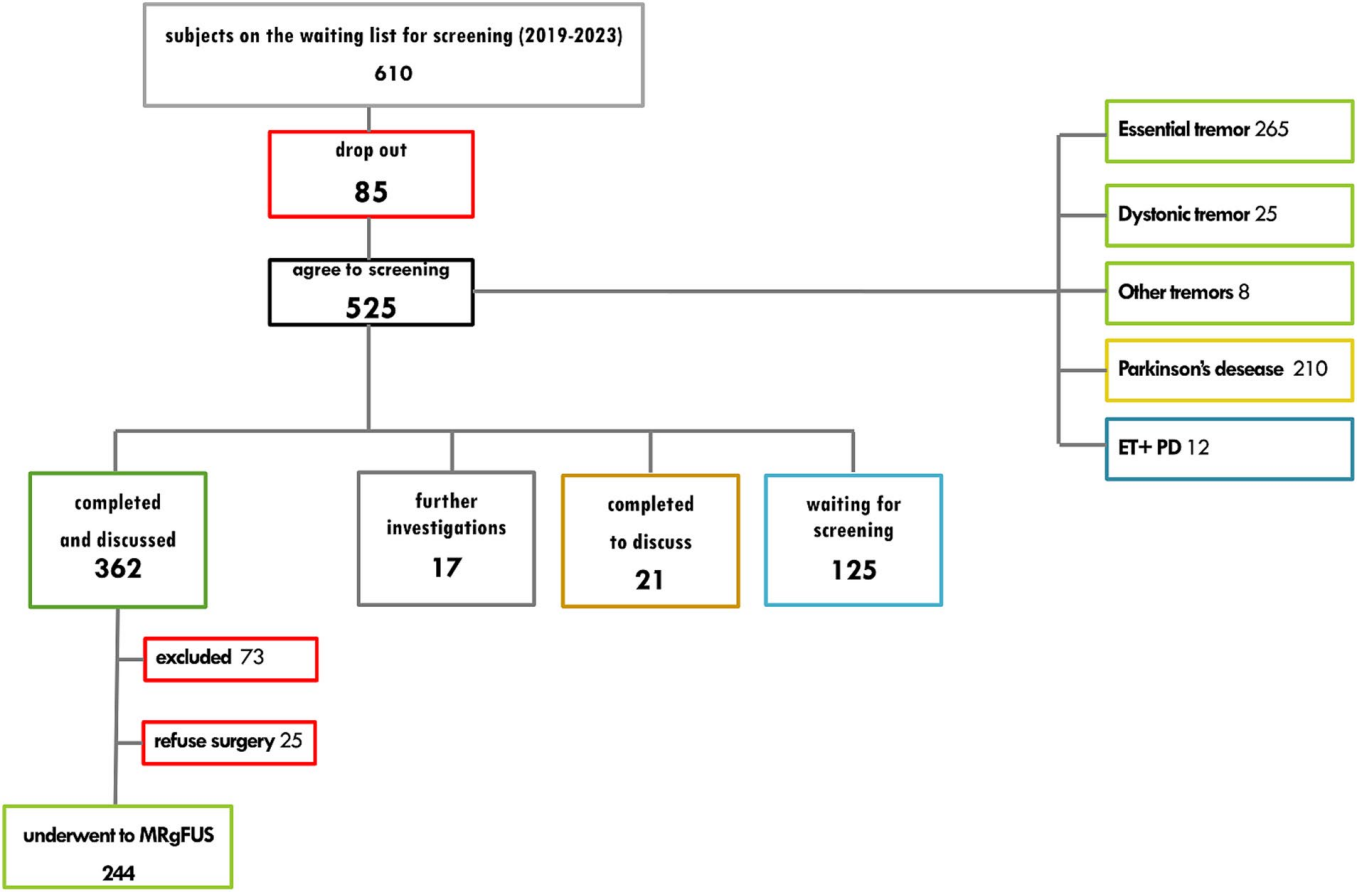
Diagnostic care pathway for the treatment of unilateral tremor with MR-guided high-field focused ultrasound: number of accesses, completed screenings and referring diagnoses.

From January 2019 to August 2023, a total of 610 patients affected by unilateral or bilateral drug-refractory tremor in individuals diagnosed with ET, dystonia, or PD, who were referred to our Institute to be screened for MRgFUS treatment. Figure 2 shows the number of accesses to the pathway, completed screenings and referring diagnoses.

**Figure 3:**
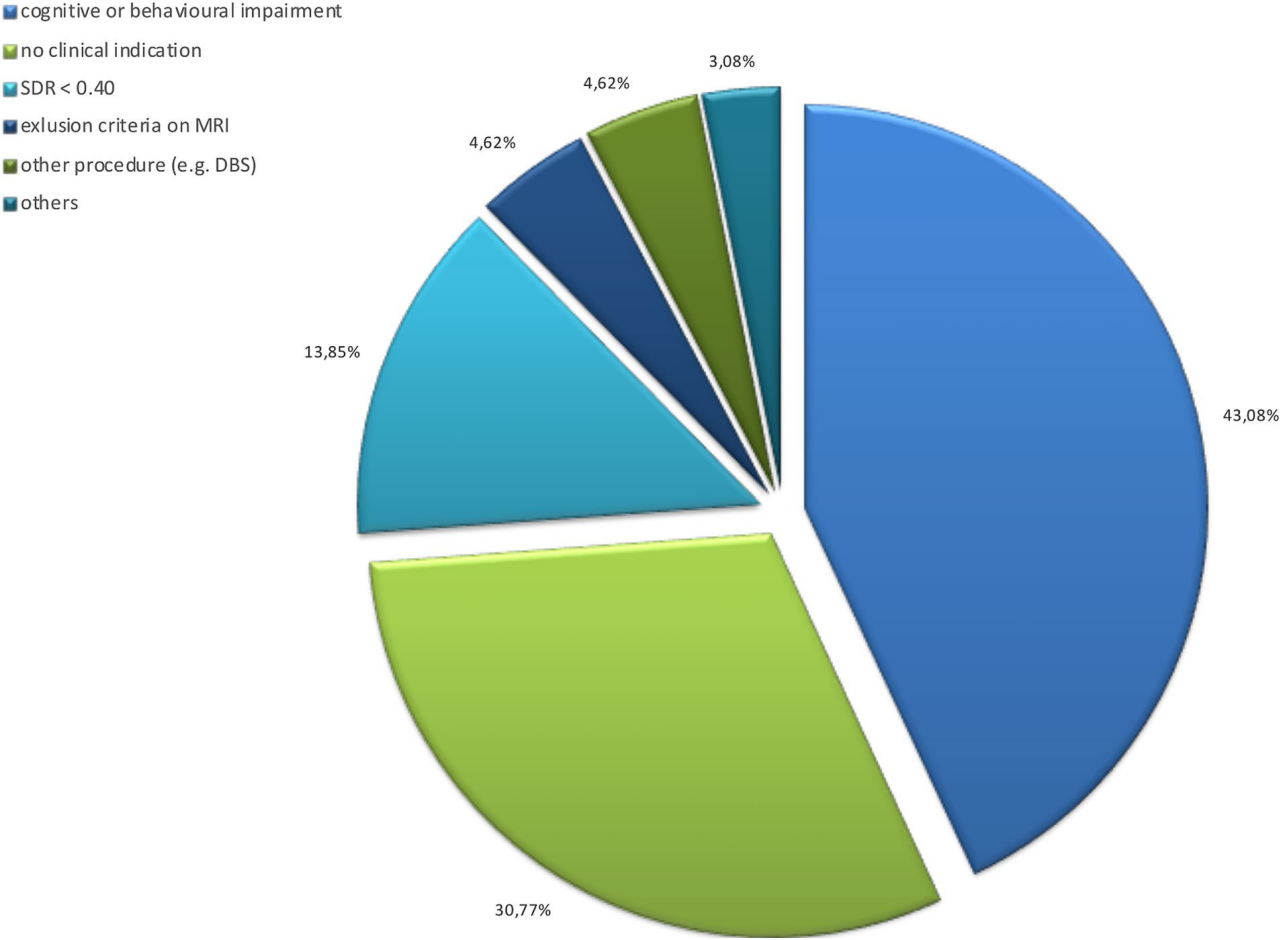
Grounds for screening failure: data on 78 assessed and excluded subjects. DBS, deep brain stimulation; SDR, skull density ratio (lower than 0.40).

Out of 362 screenings performed, 244 tested positive with indication of treatment: 25 refused the surgical procedure, 216 underwent procedure (77 for tremor in Parkinson’s disease, 139 for essential tremor). Figure 3 shows details of screening-failure results for the 73 patients that meet some exclusion criteria and did not receive indication for treatment.

Intraoperative workflow was defined as we became familiar with the use of the system (for technicians and health physicists, involved in the functional control phase of MRI and test sonication on a phantom) and the operative sequences to be performed (for radiologists), with the membrane placement phase after the stereotaxic helmet (neurosurgeons), as well as with a clinical tremor assessment system applicable in MRI (for neurologists: rest and action tremor assessment with score from 1 to 4 and paper writing tests with marker, with score from 0 to 4 for free writing, spirals, dot approximation).

After 8 months, the procedure time has been cut in half: to date, there are two procedures performed in one room session. Eighty subjects with drug-refractory diagnosed with ET or ET plus and 53 patients with tremor-dominant PD underwent the procedure.

The inpatient-surgery phase saw the initial need to initiate educational meetings with the inpatient nursing staff, who acquired basic information about the procedure and skills for managing the specific type of patient (surgical but different from oncology): despite the change of inpatient stay on 3 different operating units, due to the reorganization of beds management, there were no adverse events, near misses or sentinel events: side effects reported after the procedure are the same as those reported in the literature, with the same rates of occurrence and regression times.

The application of the PDA in the screening and follow-up phase was the phase of the course that was most informative and most evolved.

There were no major application problems, except for a start that we can describe as “uphill” due to difficulties that were not objective but related to long-standing organizational habits in the institution, which created some resistance. Outpatient activities have been acquired as a standard of care by the staff after a start-up with difficulties in assimilation and accommodation, within an old structure with few and narrow spaces whose management is not always easy: after an initial transition phase in which activities were performed at the day hospital activity area the screening visits and neuropsychological assessments were directed to the actual outpatient area with the identification of its dedicated spaces: this has made it easier for the operators at the administrative reception desk to identify individuals as outsiders and not as sent from Day Hospital and has benefited the patients, especially in the clinical follow-up phases (they already know where to go) and has avoided the continuous access of outsiders to the Day Hospital area, where they perform treatments immunocompromised patients.

The activity communication tool initially had a “chilly” reception because it appeared quite complex for some operators to understand: with a few informational and educational meetings on the subject, the interpretation issue is resolved and it is now a solid working tool. The same applies to communications regarding admissions.

Table 3 shows results for the three most significant indicators for the MRgFUS DTP.

**Table 3:** Trends for the three most significant indicators for the “DTP MRgFUS.”

	Phase 1 (selection)	Phase 2 (surgery)	Phase 3 (follow up)
2019	0.64	0.96	0.68
2020	0.61	0.97	0.97
2021	0.58	0.98	0.85
2022	0.66	0.95	0.82

Phase 1 (pre-treatment/screening): Number of cases selected for MRgFUS / number of cases proposed (total; target > 0.6). Phase 2 (treatment): Number of cases undergoing MRgFUS without complications / total number of cases treated (target > 0.85). Phase 3 (follow-up): Number of cases followed up after 1-year post MRgFUS / total number of cases treated (target > 0.6).

## Discussion

The development, implementation and evaluation of a DTP is a continuous process well represented in the Deming’s quality cycle (Plan-Do-Check-Act).

Methodically structure of all planning phases was the strategic key for the project’s success:

1. Goal setting: the definition of what, as an organization, we want to do.
2. Environmental scan: the assessments the current situation within and outside the organization by the SWOT analysis and verification of the relevance of the results of this internal/external assessment.
3. Defined strategic issues: key factors for developing an operational plan.
4. Development of new pathway and carefully organizing educational program for health professionals.
5. Defining critical success factors: achievement of objectives and implementation of strategy.
6. Development and diffusion of work plans, identification of the resources needed.
7. It was also essential to find and consider the process indicators as effective tools for providing information about the efficiency of the pathway and for adopt corrective interventions.

A coordinating/management professional was introduced for paths of high organizational complexity. This professional figure allows a reference for the definition of strategic orientations aimed at achieving a goal: to highlight the characteristics of the project and the consequent relationships with the context in which it was intended to be inserted. The resistances to development of a new pathway were mainly related to the established habit of “personalized” patient management. However, the feasibility of the pathway and the fact that the cases were already discussed at a collegial meeting made it clear to everyone that it was functional in view of the objectives.

The work plans, meeting reports, operative and discussion meetings rapidly became a solid benchmark for all the operators and operative units involved in this path.

Besides from the results obtained, the introduction of a referent for the coordination of specific DTP represented an opportunity for cultural growth in the management of the interventional therapy pathway and also for professional development. It is not easy to implement a framework aimed at sharing common protocol for screening, clinical and instrumental evaluation. The discussion of the clinical cases, to establish a joint d decision between Neurologist, Neuropsychologist, Neuroradiologist regarding the opportunity to propose the intervention, requires high competence and listening skills.

Medical doctors and staff must be completely convinced: to involve everyone and gather collaboration, it was important to get into the habit of presenting and discussing cases collegially, but also to evaluate together the basic data on the outcomes relating to the current path and communicate gradually, along the way, the clinical results for the operated patients, including the actual and practical ones resulting from the operational change. You can proceed to each discussion meeting, if you wish, with the possibility of expressing opinions and/or difficulties encountered and opening a debate on the merits.

Over time, the awareness has developed that discussing cases through discussion is a strong point: clinical cases discussion meetings have become an unmissable and rich event from a scientific point of view. The mainly practical and organizational part of the workflow is discussed during two meetings, in two key moments of the year (mid-January and early September) in which the situation is taken stock from an operational point of view.

In overcoming any obstacle to implementation, the role of the Operational Unit managers and the project contact is fundamental.

In the application of DTP, differences between the actual and reference pathways were noted, as a matter of course. These have been considered, within certain limits, “physiological” and can be generated by the specific characteristics of patients, which make each healthcare production process a singularity; a second factor considered as generating heterogeneous outcomes with respect to the reference model, are the changing operational and organizational conditions in which the provider finds itself, over time, operating. The deviations recorded, negative and positive, contributed to the refinement (design of ramifications of a basic pathway) and evolution of the reference pathway with the identification of solutions and modifications capable of generating improved results compared to the original one.

The evidences generated by the analysis of actual pathways has been the basis for rethinking the baseline pathway, suggesting the introduction of new or different activities or the elimination of activities that do not generate value (not in a strictly economic sense). Similarly, they suggested the modification of the time placement of some activities and the modulation of responsibilities in the management and delivery of other activities. In order to arrive at the analysis of deviations between reference and actual pathways, it was essential to undertake a focused study of the care pathway, describing its salient points in detail in some respects but without presumption of exhaustiveness in other respects. The identification of the activities that make up the patient’s overall care pathway and that contribute, in a coordinated and finalized form, to the resolution of a need. They have different natures (clinical, care, social, environmental, supportive, direct, indirect, etc.) and can be the most diverse, depending on the specific needs and the institutional entity in charge of them.

Knowing what is carried out during a health care process can lead to questions about how and why certain activities are delivered. Fundamental is to observe how activities are combined, how the organization makes them available, at what times and in what places, and whether with the integration of the different units participating in the overall process. Described the “production” process, in terms of combined activities, the critical activities highlighted in the overall process are highlighted and discussed, making it possible to evaluate production and delivery alternatives.

Some critical issues remain unresolved related to the limited resources available and how/who to involve. In this regard, we are evaluating some possible organizational changes that will allow the project to be more sustainable.

The MRgFUS DTP operating model was adopted as the basis for all complex diagnostic outpatient pathways initiated at the Institute. For interventional therapies, we completed the mapping of the diagnosis and treatment process “Interventional Therapies Movement Disorders” by extending the application of the “model-MRgFUS” to other interventional therapies as well.

## Data availability statement

The datasets presented in this article are not readily available because of ethical and privacy restrictions. Requests to access the datasets should be directed to the corresponding author.

## Ethics statement

Written informed consent was obtained from the individual(s) for the publication of any potentially identifiable images or data included in this article.

## Author contributions

SR: Conceptualization, Data curation, Investigation, Methodology, Project administration, Writing – original draft, Writing – review & editing. RC: Methodology, Supervision, Writing – review & editing. VaL: Writing – review & editing. MGa: Writing – review & editing. NA: Writing – review & editing. FC: Writing – review & editing. AB: Writing – review & editing. RT: Writing – review & editing. MP: Writing – review & editing. ViL: Writing – review & editing. LR: Writing – review & editing. AE: Writing – review & editing. FG: Writing – review & editing. EM: Writing – review & editing. MF: Writing – review & editing. FE: Writing – review & editing. SaP: Writing – review & editing. PA: Writing – review & editing. SyP: Writing – review & editing. GD: Writing – review & editing. VN: Writing – review & editing. EC: Writing – review & editing. MS: Writing – review & editing. MGr: Writing – review & editing. VC: Writing – review & editing. MC: Writing – review & editing. FD: Writing – review & editing. GM: Writing – review & editing. RE: Formal analysis, Funding acquisition, Methodology, Project administration, Resources, Supervision, Validation, Writing – review & editing.
